# Molecular Insights into Crimean-Congo Hemorrhagic Fever Virus

**DOI:** 10.3390/v8040106

**Published:** 2016-04-21

**Authors:** Marko Zivcec, Florine E. M. Scholte, Christina F. Spiropoulou, Jessica R. Spengler, Éric Bergeron

**Affiliations:** Viral Special Pathogens Branch, Division of High Consequence Pathogens and Pathology, Centers for Disease Control and Prevention, Atlanta, GA 30333, USA; mzivcec@cdc.gov (M.Z.); kyj7@cdc.gov (F.E.M.S.); ccs8@cdc.gov (C.F.S.); wsk7@cdc.gov (J.R.S.)

**Keywords:** Crimean-Congo hemorrhagic fever, viral hemorrhagic fever, reverse genetics, pathogenesis, tick-borne virus

## Abstract

Crimean-Congo hemorrhagic fever virus (CCHFV) is a tick-borne pathogen that causes high morbidity and mortality. Efficacy of vaccines and antivirals to treat human CCHFV infections remains limited and controversial. Research into pathology and underlying molecular mechanisms of CCHFV and other nairoviruses is limited. Significant progress has been made in our understanding of CCHFV replication and pathogenesis in the past decade. Here we review the most recent molecular advances in CCHFV-related research, and provide perspectives on future research.

## 1. Introduction

Crimean-Congo hemorrhagic fever virus (CCHFV) causes a mild to severe hemorrhagic disease (CCHF) exclusively in humans, with case fatality rates of 5%–30%. Presently, efficacy of therapeutic options in controlled clinical trials remains unproven, and supportive care remains the mainstay of treatment. CCHF endemic foci are present over a wide geographic range, including areas in Western and Central Asia, the Middle East, South-Eastern Europe, and Africa [1,2,3,4]. CCHFV exists in an enzootic cycle between ticks and mammals; and geographic distribution of the virus mirrors the distribution of the primary tick vector species that include members of the *Hyalomma* genus (*H. marginatum*, *H. anatolicum*, *H. truncatum*, *H. impeltatum*, and *H. impressum*) [5]. Viral transmission to humans can occur via tick bite, or via exposure to body fluids from viremic animals or humans [3].

Following a brief incubation period (usually <7 days), CCHFV infection initially causes non-specific symptoms, including a rapid onset high-grade fever, fatigue, and myalgia, frequently accompanied by vomiting and diarrhea. Progression to severe disease is characterized by thrombocytopenia, elevated circulating liver enzymes, and hemorrhagic manifestation (petechiae, ecchymosis, and epistaxis, as well as gingival, gastrointestinal, and cerebral hemorrhages) [1,2,3,4]. Severity of CCHF correlates with increased viral load and dissemination, low anti-CCHFV antibody titers, severity of thrombocytopenia, increased clotting times, hemorrhage, high levels of pro-inflammatory cytokines (e.g., tumor necrosis factor α (TNFα), interleukin 6 (IL-6)), and elevated aspartate aminotransferase and alanine aminotransferase [6,7,8,9,10,11,12,13,14,15,16,17,18]. Fatal outcome is typically the result of disseminated intravascular coagulopathy, shock, and/or multi-organ failure [1,2,3,4,19]. During the course of disease, CCHFV is widely distributed throughout the body, and has been detected in spleen, lung, heart, and intestinal tissues in fatal human cases [19]. The main cellular targets of infection are mononuclear phagocytes, endothelial cells, and hepatocytes [19]. Infection of monocyte-derived macrophages, endothelial cells, and dendritic cells are confirmed *in vitro* [20,21,22].

Continued research into CCHF and the development of medical countermeasures is needed based on severity of disease, human-to-human transmission, the absence of vaccines or treatments with proven efficacy and the potential for a severe outbreak in the future. Although research on CCHFV is limited, there have been significant recent advances in CCHFV research. These include modern molecular tools and *in vivo* disease models that offer opportunities for substantial progress in the field, and further development of therapeutics and vaccines. In this article, we summarize current knowledge of CCHFV genome replication, viral protein processing and function, and the development of reverses genetic systems. We also highlight areas of importance for future research.

## 2. CCHFV Genome and Replication Cycle

### 2.1. CCHFV Genome

**Figure 1:**
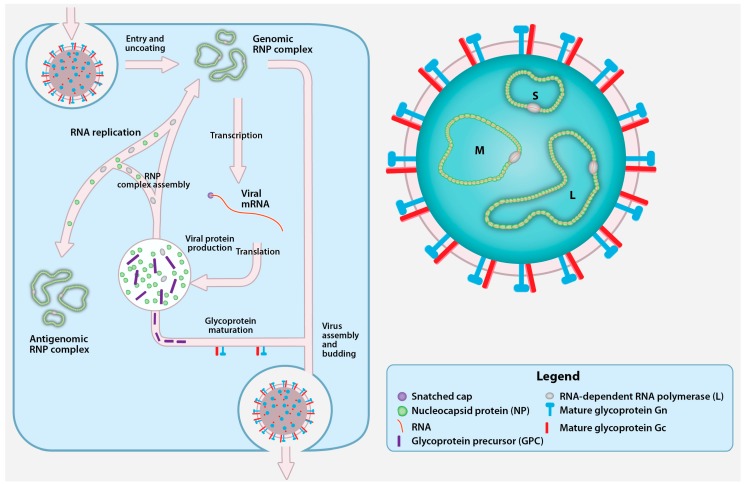
Crimean-Congo hemorrhagic fever virus (CCHFV) virion and replication cycle. The CCHFV virion contains a tri-segmented, negative-sense, single-stranded RNA (vRNA) genome encapsidated by the nucleoprotein (NP) and the RNA-dependent RNA polymerase (RdRp; L protein). Together, vRNA, NP, and RdRp form the genomic ribonucleoprotein complexes (RNP) inside a cellular membrane-derived envelope coated with the mature glycoproteins Gn and Gc. CCHFV attaches to an unidentified cellular receptor and enters the cells in a clathrin-dependent manner. After fusion with the cellular membrane, the viral genomic segments are uncoated and transcribed by L protein into viral mRNA that gain host cell-derived 5′ caps by cap snatching. The viral mRNAs are translated into the NP and L proteins by cytoplasmic ribosomes, while the glycoprotein precursor (GPC) appears to be translated by endoplasmic reticulum (ER)-associated ribosomes. A portion of the newly synthesized NP and L protein are used to replicate the genomic RNA by forming an RNP containing antigenomic RNA (cRNA). The GPC undergoes processing and maturation in the ER and the Golgi, and yields the Gn and Gc. Upon the accumulation of nascent mature glycoproteins and genomic RNPs, new CCHFV particles assembly is believed to occur in the Golgi followed by virion release in Golgi-derived vesicles.

CCHFV is a member of the genus *Nairovirus* in the family *Bunyaviridae*, which includes five genera and over 350 virus species [23]. CCHFV is characterized by a tripartite RNA genome of negative polarity (Figure 1), and by tick-borne maintenance and transmission. Complementary non-coding regions (NCRs) are present at the 5′ and 3′ termini of the small (S), medium (M), and large (L) segments of CCHFV and contribute to the circular appearance of the bunyavirus genomes [24]. The nine terminal nucleotides (5′-UCUCAAAGA and 3′-AGAGUUUCU) are conserved between nairoviruses, and serve as viral promoter regions. The NCRs are necessary for the viral RNA-dependent RNA polymerase (RdRp or L protein) to bind and initiate transcription and/or replication of the viral genome [25,26,27]. Although the complete NCRs sequences differ between viral segments, each is capable of initiating encapsidation, transcription, replication and packaging of the genomes into nascent virions.

**Figure 2:**
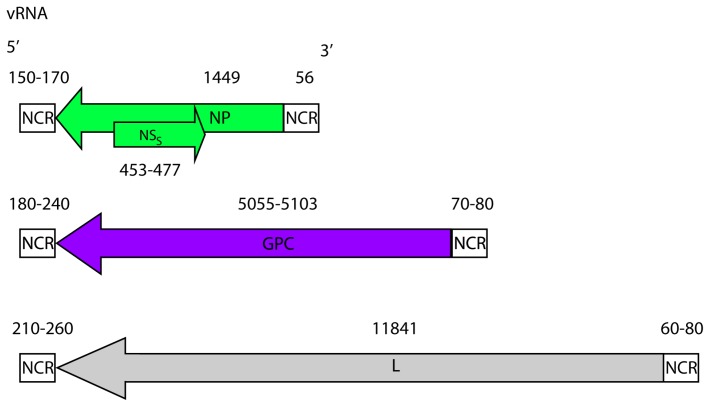
CCHFV genome. CCHFV possess a tri-segmented negative sense RNA genome. The small (~1.6 kb), medium (~5.4 kb) and large (~12.1 kb) segments, code for the NP, the GPC and the L protein, respectively. The small segment also codes for a non-structural S protein (NS_S_) in the positive sense. The coding regions are flanked by non-coding regions (NCRs). The nucleotide lengths of the regions (both coding and non-coding) are displayed and based on full-length sequences available in GenBank.

Until recently, all three segments of CCHFV were believed to each encode a single protein. However, a second protein, the non-structural S (NS_S_), is encoded in the S segment in the opposite orientation relative to the nucleoprotein (NP) gene, indicating that CCHFV might be considered an ambisense virus (Figure 2) [28]. However, the ambisense coding in CCHFV involves overlapping coding regions. This differs from ambisense coding in bunyaviruses and arenaviruses where the viral proteins are encoded in opposite orientation, and separated by an intergenic region that serves as a transcription termination signal [29,30]. In contrast to the S segment (~1.6 kb), that is comparable in size to other bunyaviruses, the M (~5.4 kb) and L (~12.1 kb) segments of CCHFV are significantly larger than those of other bunyaviruses, and contain a single gene encoding the glycoprotein precursor (GPC) and the L protein, respectively.

The genome segments or viral RNA (vRNA) are encapsidated with NP and L protein to form genomic ribonucleoprotein complexes (RNP). The genomic RNPs are packaged into viral particles by acquiring a lipid envelope and the surface glycoproteins Gn and Gc [31] (Figure 1). Electron microscopy studies of nairoviruses report spherical particles of relatively uniform size with a diameter of ~90–100 nm with small (<10 nm) spike-like projections from the surface of the particle [27,32,33,34,35,36]. Further characterization of the precise arrangement of the surface glycoproteins will require more refined methods such as cryo-electron microscopy.

### 2.2. Cell Entry

The initial binding of CCHFV to the cell surface is mediated by the glycoproteins Gn and/or Gc. However, the details of specific glycoprotein involvement in viral attachment, internalization, and fusion remain unknown. It is suspected that Gc is responsible for binding to the cellular receptors, as monoclonal antibodies targeting Gc can neutralize CCHFV infection of mammalian cells [37]. The Gc of CCHFV is also thought to mediate fusion; the predicted fusion loop of CCHFV [38] shares significant homology with Rift Valley fever virus (RVFV) Gc fusion loop [39]. The cellular receptors required for CCHFV entry have not been identified. A functional interaction has been suggested between CCHFV Gc and cell surface nucleolin, a protein found predominantly within nucleoli [40]. Nucleolin serves as a receptor for the respiratory syncytial virus, functions as an entry factor for Japanese encephalitis virus, and enhances entry of human immunodeficiency virus (HIV) [41,42,43,44]. However, further investigations in the context of CCHFV infection are needed to support the involvement of nucleolin in CCHFV cell binding and/or fusion.

After attaching to the cell surface, CCHFV is endocytosed through a clathrin-mediated endocytosis mechanism. Entry requires clathrin and the clathrin pit adaptor protein-2 complex, but not caveolin-1 [45,46,47]. CCHFV entry is also dependent on cholesterol and a low pH [45,46]. Following endocytosis CCHFV particles are transported to early endosomes and subsequently to multivesicular bodies (MVB) in a process that is dependent on Rab5 [46,47].

In contrast, blocking Rab7-dependent trafficking (from early endosomes to the late endosomes, or transport out of the MVB) has no effect on infection or CCHFV association with MVB [47]. Interfering with the formation of functional MVBs, for example, by depleting components of the endosomal sorting complex required for transport (ESCRT) pathway decreased CCHFV infection levels [47]. This indicates that the MVB is likely the main organelle where the CCHFV envelope fuses with the host membranes.

### 2.3. Transcription and Replication

Following cell entry and fusion, the genomic RNPs are released into the cytosol and the encapsidated vRNA serves as a template for the L protein to synthesize viral mRNA (Figure 1). No studies have described the 3′ termini of nairovirus mRNA or elements involved in terminating transcription. However, the 5′ terminal regions of Dugbe virus, a related nairovirus, contain a 7-methylguanylate (m7G) cap with sequences derived from cellular mRNA. To initiate viral mRNA synthesis, the L protein uses m7G capped primers that are snatched from cellular mRNA by an endonuclease domain located in the L protein. CCHFV L protein contains a residue (D693) [25] that is predicted to coordinate a Mn^2+^ critical for the cap snatching activity, as demonstrated for endonucleases of other L proteins [48,49,50]. Mutating D693 selectively abolishes L protein transcription activity, but does not impair its ability to replicate CCHFV genome analogues [26]. This suggests that capped primers are not used to initiate CCHFV replication.

The replication of genomic RNPs is a process requiring the replication and encapsidation of uncapped, negative sense vRNA and positive sense complementary RNA (cRNA). The replication of the RNPs minimally require the L protein and NP [26]. The encapsidated forms of the cRNA and vRNA are respectively defined as antigenomic and genomic RNPs (Figure 1). Since CCHFV is a negative strand RNA virus, genomic RNPs are used as template to synthesize capped mRNA and produce antigenomic RNPs. During the replication of the genomic RNPs, a cRNA is synthesized by the L protein and NP subunits are added to the elongating strands to obtain antigenomic RNPs, and in turn the cRNA of the antigenomic RNPs is used a template to obtain genomic RNPs.

### 2.4. Glycoprotein Maturation, Viral Assembly, and Egress

**Figure 3:**
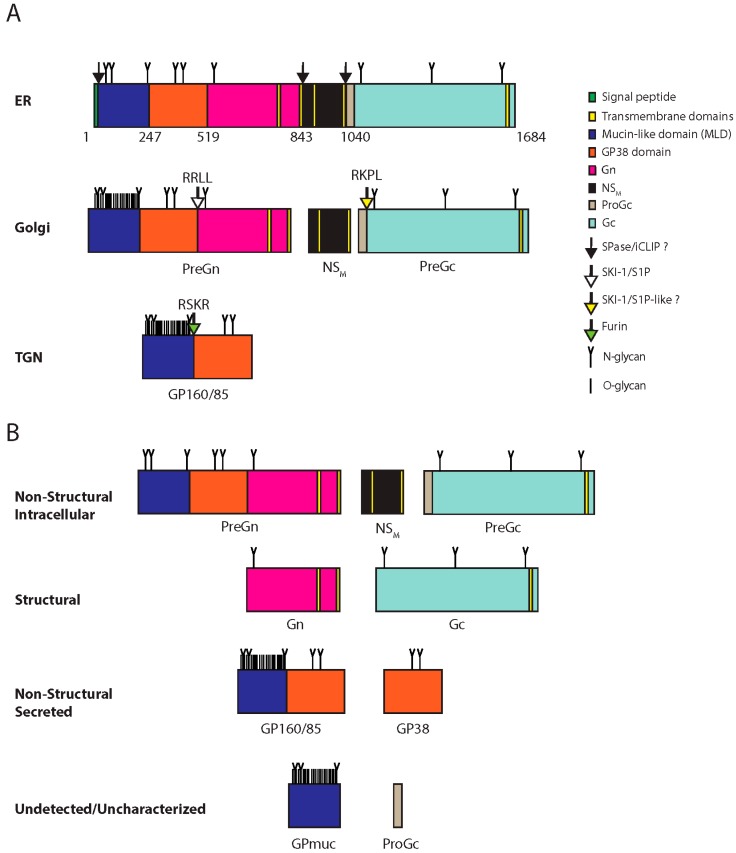
CCHFV glycoprotein processing and products. (**A**) CCHFV glycoprotein processing. The GPC is synthesized in the ER where *N*-glycosylation occurs (*N*-glycan). Numbers indicate amino acids positions. The signal peptidase and/or the intramembrane cleaving proteases (iCLIPs) co-translationally cleave GPC close to or within transmembrane domain-2 and -4. These cleavages yield PreGn, non-structural M protein (NS_M_) and PreGc. These proteins traffic to the Golgi where the mucin-like domain of PreGn is *O*-glycosylated (*O*-glycan), and is cleaved by subtilisin kexin isozyme-1/site-1 protease (SKI-1/S1P) at the RRLL motif. PreGc at the RKPL motif by a protease with similar specificity to SKI-1/SIP (SKI-1/S1P-like?). PreGn cleavage liberates an N-terminal fragment with an apparent total molecular weight on SDS-PAGE of 160 kDa (GP160) and 85 kDa (GP85). GP160/85 is later cleaved by furin in the *trans*-Golgi network (TGN). (**B**) GPC products. Processing of GPC yields non-structural products associated with the cell (Non-structural intracellular), associated with the virions (Structural), secreted but not part of the virions (Non-structural secreted), and inferred products that remain uncharacterized or have yet to be detected Undectected/uncharacterized).

RNP replication is followed, and later accompanied, by viral protein processing and subsequent maturation. The maturation of CCHFV GPC is unusually complex and shares little similarity with glycoprotein processing in other bunyaviruses [31]. GPC maturation yields the structural glycoproteins Gn and Gc; secreted non-structural proteins GP160, GP85, and GP38 [31,51]; and the non-structural M protein (NS_M_) [52] (Figure 3). Nairoviruses are the only bunyaviruses known to encode secreted non-structural glycoproteins [31,37,52,53,54,55,56,57]. To yield the complete set of glycoproteins, the GPC is heavily glycosylated [31] and subsequently cleaved by host proteases including the proprotein convertases [51,54,56,58], a family of mammalian serine proteases known to process a variety of cellular proteins, viral glycoproteins, and bacterial toxins [59].

The GPC contains an N-terminal signal peptide directing its synthesis to the secretory pathway [31,52]. Upon elongation and translocation into the endoplasmic reticulum (ER), the signal peptide is removed, the GPC is *N*-glycosylated, folded, intra-molecular disulfide bridges are formed, and the transmembrane domains of the precursor protein span the ER membrane five times [31]. Before GPC is completely translated, it is cleaved into the Gn precursor (PreGn), the Gc precursor (PreGc), and the NS_M._ This step is thought to require the signal peptidase and the intramembrane cleaving proteases (I-CLiPs), as generation of these three proteins requires cleavage after the signal peptide and near or within the transmembrane domains-2 and -4 (Figure 3) [31,52].

The productive maturation in the ER is followed by PreGn and PreGc transport to the Golgi complex [52,54], where the PreGn mucin-like domain (MLD) acquire numerous *O*-linked glycans [51]. PreGn can traffic to the Golgi complex, the proposed site of CCHFV assembly, in the absence of PreGc [37], but PreGc requires PreGn to exit the ER [37]. Mutating the N577 glycosylation site of PreGn, located in the mature Gn region, blocks PreGn from exiting the ER and prevents secretion of GP160, GP85, and GP38, suggesting a critical role for the glycosylation of N577 in folding and normal trafficking to the Golgi complex [55].

Limited endoproteolysis PreGn and PreGc is required to complete GPC maturation. The host protease required to convert PreGc to Gc remains unidentified. Given that the PreGc cleavage motif of CCHFV (RKPL) is identical to the GPC cleavage motif of Guanarito virus, an arenavirus [60] that is cleaved by the subtilisin kexin isozyme-1/site-1 protease (SKI-1/S1P), this same protease or a protease with similar specificity (SKI/S1P-like) may cleave CCHFV PreGc. The cleavage of PreGn by SKI-1/S1P occurs early in the secretory pathway, either in the ER or after its exit to the *cis*-Golgi apparatus [58]. PreGn cleavage at the RRLL motif liberates N-terminal products (GP160 and GP85), containing the MLD and GP38 domain [58]. GP160/85 can be further cleaved in the *trans*-Golgi network (TGN) by furin at a well-conserved RSKR motif located at the junction of the MLD and the GP38 domain [51]. This cleavage is predicted to free the GP38 domain from the MLD, although available antibodies can only detect GP38 and the uncleaved MLD-containing glycoproteins (GP160/85), but not the cleaved MLD.

Initial speculation was that GP160 was a dimer of GP85 because epitopes located within the MLD and GP38 domains are present in both glycoproteins, but denaturation with urea and reducing agents did not alter the mobility of GP160 or GP85. Additionally, the glycan composition of the non-structural glycoproteins (GP160, GP85 and GP38) is likely similar based on comparable sensitivity to glycosidases specific for *N*- and *O*-linked glycans [31,51]. Therefore, additional experiments are needed to explain the difference in the mobility of GP85 and GP160 on SDS-PAGE.

Our understanding of CCHFV assembly and egress is limited. Like other bunyaviruses, CCHFV RNPs are most likely found in the cytoplasm, and NP is localized in the perinuclear region close to the Golgi complex [61]. The subcellular localization of the viral glycoproteins often dictates the location of virus budding; the accumulation of the glycoproteins in the Golgi complex and TGN [37,54] and NP in perinuclear regions is compatible with budding and assembly of CCHFV particles in the Golgi complex and/or TGN. [61,62]. Following assembly, CCHFV particles are released by exocytosis often in the absence of discernable cytopathology and egress occurs from the basolateral membrane in polarized epithelial cells [63].

## 3. Viral Protein Function

### 3.1. S Segment: NP and NS_S_

The primary function of NP is to encapsidate the vRNA and cRNA to form RNPs. Consistent with its role in RNP formation, NP has been shown to interact with the N- and C-terminal regions of the L protein and to co-localize with tagged L protein in transfected cells [26,64]. L protein antibodies are currently unavailable for CCHFV, preventing the study of L protein in infected cells. In CCHFV-infected cells, NP is localized to the perinuclear region in an actin filament-dependent manner, and interfering with actin polymerization reduces CCHFV infectivity [63]. NP likely performs multiple other functions. For example, NP can be released from cells even in the absence of other viral particles and leads to the formation of spherical particles resembling bunyaviruses, suggesting a possible role in viral budding [32].

Important insights into the roles of NP were obtained from the crystal structures of CCHFV NP using CCHFV isolates from Iraq [65] and China [66,67]. The protein structure is more closely related to the NP of arenaviruses [68,69] than to that of NP of other bunyaviruses [70,71,72]. The NP possesses two major domains: a globular head and a flexible arm. The arm domain protrudes from the globular head domain of CCHFV NP. The globular head domain of CCHFV NP comprises 23 α-helices with an overall structure that is similar to the NP of Lassa virus (LASV), an arenavirus. Crystallization of NP as an oligomer in the presence of RNA demonstrated that NP subunits organize in a head-to-tail orientation, with the arm of one subunit interacting with the head domain of the neighboring subunit, that are further assembled into double antiparallel superhelical polymers of NP; this may represent the organization of NP in the RNPs [73].

Oligomerization appears to regulate NP function. In the absence of RNA, NP appears to exist exclusively as a monomer; in this state, NP binds RNA weakly [66,67], suggesting that only NP oligomers effectively bind RNA. Additionally, comparing monomeric and oligomeric NP organization shows that the arm domain changes conformation upon oligomerization allowing stronger binding to RNA [73]. The nature of the chemical bonds between RNA and NP remain unknown, as the RNA structure has yet to be resolved in any of the NP models. However, mutating three CCHFV NP residues (K132, Q300, K411) predicted to bind RNA blocked the transcription and replication of CCHFV minigenomes [65,67].

NP may contribute to viral-mediated immune evasion. Multiple aspects of the mammalian antiviral response appear to target the NP, like the interferon (IFN)-stimulated antiviral gene MxA, which inhibits CCHFV replication [62]. Furthermore, NP is highly immunogenic and is the major target of both B and T cells in mammals [37,74,75,76,77]. The NP of LASV and related hantaviruses have been shown to function as IFN antagonists [69,78]. However, CCHFV NP does not suppress the IFN response to Sendai virus infection [67], a model virus that strongly activates the innate immune responses to double stranded RNA (dsRNA). Structurally related arenavirus NPs possess a conserved 3′ to 5′ exonuclease activity specific to RNA, and this RNase activity suppresses the innate immune response to dsRNA [69]. *In vitro*, CCHFV NP has endonuclease activity, but appears to be restricted to DNA instead of RNA [67].

Early in infection CCHFV can inhibit apoptosis [79], but later in infection it induces apoptosis [80]. During apotosis, a fraction of NP is cleaved by caspase-3 at a putative DEVD motif [80] located in the apex of the arm domain. Since the arm domain interacts with the head domain in NP oligomers, steric hindrance associated with NP oligomerization would prevent cleavage of NP by caspase-3 [73]. NP monomers may prevent apoptosis by acting as decoy substrate for caspase-3 to delay apoptosis as suggested for a structurally related arenavirus, Junín virus [81].

In transfected cells, the recently described CCHFV NS_S_ localizes to the mitochondria and induces apoptosis by disrupting the mitochondrial membrane potential [28]. Although NS_S_ has been detected in CCHFV-infected cells, the proposed apoptotic functions are based exclusively on the overexpression of NS_S_, and further investigations of NS_S_ are warranted.

### 3.2. M Segment: Glycoproteins

Overall, CCHFV glycoproteins appear to be involved in entry and fusion, virion formation and immune evasion. Gn and Gc are believed to mediate entry and fusion. Their functions are for the most part extrapolated from those of glycoproteins from distantly related bunyaviruses. The cytoplasmic tail of Gn contains a zinc finger domain that binds RNA in vitro, suggesting that Gn may interact with RNA and perform matrix protein-like functions [53]. However, as RNA [3] is encapsidated in the RNP, it is not clear how Gn might bind and incorporate RNP into the nascent virions. Functional studies of the CCHFV GPC highlighted the essential role of the PreGn convertase SKI-1/S1P in the production of infectious particles [54]. Importantly, PreGn and PreGc normally localize in the Golgi complex, which implies that correct processing of these proteins regulates the production of infectious CCHFV particles [54]. Determining the function of GP38 is particularly intriguing as it is nairovirus-specific and does not share sequence homology with other cellular or viral proteins. Furin cleavage was shown to selectively block GP38 production without affecting the secretion of the cleavage products of SKI-1/S1P: GP160 and GP85 [51,56]. Blocking furin cleavage resulted in a transient reduction in viral titers, implying that mature GP38 is not important for CCHFV replication, at least in cell culture. In addition, blocking furin cleavage also results in a slight reduction in PreGn processing, showing that furin cleavage might indirectly regulate SKI-1/S1P-dependent GPC processing [56].

The MLD is found in PreGn, GP85, and GP160. Interestingly, the glycoprotein precursors of filoviruses (e.g., Ebola virus and Marburg virus) also contain a MLD and a furin cleavage site [82,83]. In filoviruses, furin cleavage products, GP1 and GP2, are structural components of virions, and the O-linked glycans of the GP1 MLD can shield and protect GP2 epitopes targeted by neutralizing antibodies [84]. The Ebola virus MLD can be replaced with the CCHFV MLD without affecting protein function, suggesting that the MLD of CCHFV may also shield exposed epitopes [84]. However, the function of the CCHFV MLD may differ significantly from that of filoviruses, as the CCHFV MLD is not incorporated into the viral particles [31]. In addition, truncating the CCHFV MLD is dispensable for the folding and trafficking of GPC [37]. In contrast, longer N-terminal truncation comprising the GP38 domain retains Gn in the ER, suggesting that the GP38 domain has a chaperone activity that assists PreGn folding or frees it from the ER [37].

### 3.3. L Segment: Ovarian Tumor Protease, Nuclease, and RdRp Activities

**Figure 4:**
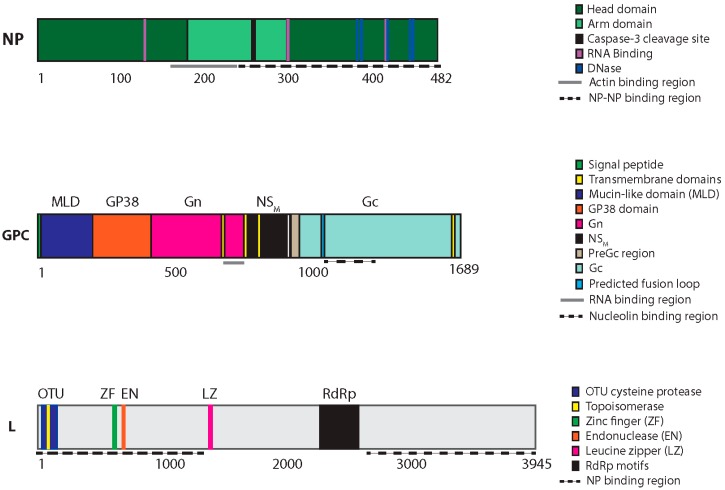
CCHFV protein domains, motifs and catalytic residues. The three CCHFV genomic segments (S, M and L) are translated into three proteins: NP, the GPC, and the L protein, respectively. The GPC is proteolytically processed to yield several additional proteins, including Gn and Gc. The approximate total size and location of motifs and catalytic residues of each protein is indicated below in amino acids.

Nairoviruses have an unusually large L protein (~4000 amino acids) compared to those of other family *Bunyaviridae* members (~2500 amino acids). Additional sequences are present in the N-terminal region of nairovirus L proteins that are not found in other *Bunyaviridae* genomes. This finding suggests that amino acids 1–609 may contain domains with non-classical L function, such as the ovarian tumor (OTU) cysteine protease. Sequences located between the OTU domain and the RdRp conserved motifs contain a potential leucine zipper and a C2H2 zinc finger motif important for binding NP the N-terminal region of the L protein [64,85] (Figure 4).

The L protein regions involved in mRNA transcription and replication of the viral genome likely start with the internal endonuclease domain and include several conserved RdRp motifs [85,86]. The viral endonuclease cleaves host mRNAs and uses the resulting 5′ capped oligonucleotides as primers to initiate viral transcription. The 5′ termini of CCHFV vRNA are monophosphorylated [87,88], in contrast to many other RNA viruses that use the more common triphosphate group (5′-pppRNA). Monophosphorylated 5′ genome ends are likely created by a chain initiation mechanism called prime and realign, in which the viral endonuclease generates a 5′-pRNA by cleaving off the first nucleotide of the 5′ genomic end. This mechanism was previously suggested for the related Hantaan virus [89]. Processing of CCHFV genome 5′ termini to a monophosphate group (5′-p) is a possible strategy for evading the innate immune response by blunting the activation of retinoic acid-inducible gene I (RIG-I), which is preferentially activated by 5′-pppRNA [87,88]. Nevertheless, the type-I IFN response to CCHFV requires RIG-I [88]. RIG-I is believed to function not only as a sensor during viral infection, but also as an antiviral effector [90]. This effector function may partially explain why RNA viruses that induce poor IFN responses or do not have the preferred 5′-pp or 5′-pppRNA RIG-I ligands replicate more efficiently when RIG-I is knocked down [88,91].

The most extensively studied region of the CCHF L protein is the N-terminal OTU domain (residues 1–152). The OTU domain removes ubiquitin (Ub) and Ub-like protein IFN-stimulated gene-15 (ISG15) from their protein substrates [92,93,94]. Viral OTUs and papain-like proteases with similar activity have previously been found in both positive- and negative-stranded RNA viruses, including Dugbe virus, Nairobi sheep disease virus, rice stripe virus, porcine reproductive and respiratory syndrome virus, equine arteritis virus, murine hepatitis virus, severe acute respiratory syndrome coronavirus, human coronavirus NL63, and Middle East respiratory syndrome coronavirus [86,92,94,95,96,97,98,99,100,101]. In addition, deubiquitinases of positive-strand RNA viruses proteolytically process the viral polyproteins and are therefore necessary for replication [94]. In CCHFV, the L protein is not proteolytically processed by the OTU domain, and the OTU cysteine protease activity is dispensable for CCHFV transcription and replication of minigenomes [26].

Mammalian deubiquitinases are implicated as a negative feedback system of the IFN response [102], and viral OTUs appear to perform similar functions. The CCHFV OTU domain is thought to suppress innate immune signaling by deconjugating Ub or ISG15. Conjugation of Ub (ubiquitination) and ISG15 (ISGylation) to lysine residues regulates IFN signaling, and targets several key components of the innate immune response, including nuclear factor kappa-light-chain-enhancer of activated B cells (NFκB), RIG-I, MxA, interferon regulatory factor 3 (IRF3), signal transducer and activator of transcription 1 (STAT1), and protein kinase R (PKR) [103,104,105,106,107,108]. The crystal structure of CCHFV OTU domain provided insights into Ub and ISG15 binding specificity and allowed the design of CCHFV mutants specifically lacking activity against Ub or ISG15 [93,109,110]. Overexpression of the CCHFV OTU domain results in reduced general cellular ubiquitination and ISGylation levels. In addition, CCHFV OTU overexpression reduces the RIG-I/mitochondrial antiviral-signaling protein (MAVS)-mediated IFN-response, likely because this overexpression blocks ubiquitination of RIG-I [92,94]. The putative role of the OTU in infection remains paradoxical, as ubiquitination of RIG-I by tripartite motif-containing protein 25 (TRIM25) activates it, whereas ISGylation of RIG-I negatively regulates RIG-I activation by competing with ubiquitination sites [103,111]. More studies are required to understand how deubiquitination of RIG-I by a viral OTU may facilitate viral replication, while simultaneous removal of ISG15 moieties from RIG-I may also result in increased antiviral responses.

## 4. CCHFV Reverse Genetics

CCHFV reverse genetics systems are powerful tools for investigating underlying mechanisms of the viral replication cycle, host immune evasion, and numerous other pathogen-host interactions. These systems are particularly useful for dissecting and studying the basics of the viral replication cycle, including viral genome transcription, replication, particle assembly and egress. In addition, reverse genetics enables the generation of reporter viruses and allows mutational analyses, for example when studying viral drug resistance or pinpointing catalytic residues.

### 4.1. CCHFV Minigenome System

**Figure 5:**
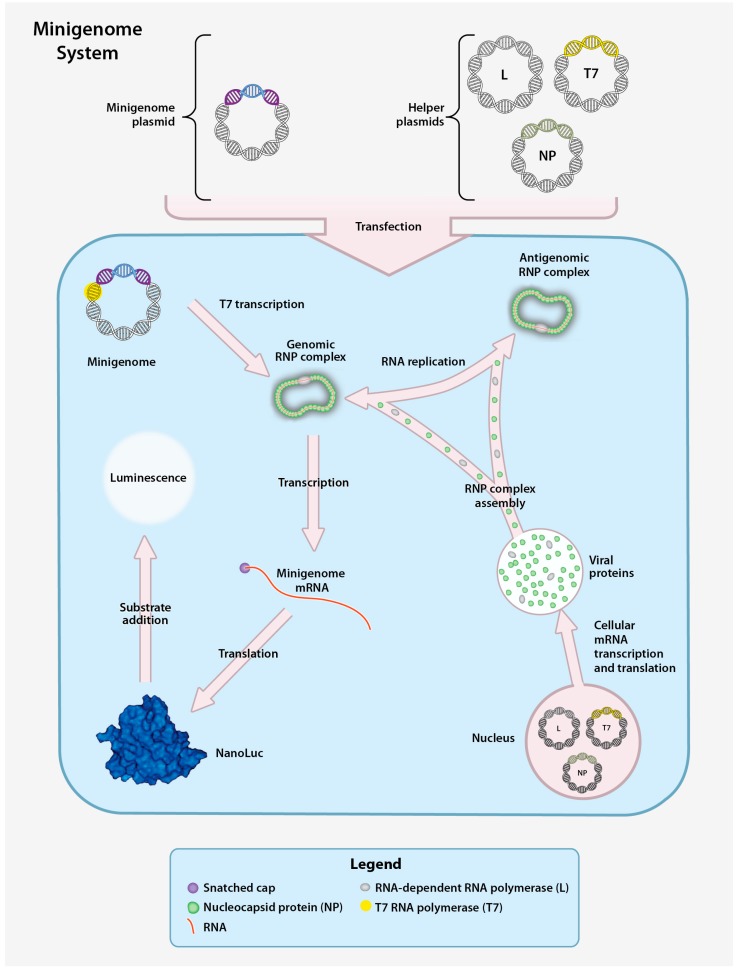
CCHFV minigenome system. The CCHFV minigenome system is composed of a plasmid encoding the minigenome and three helper plasmids encoding the CCHFV NP, L, and T7 RNA polymerase (T7) genes downstream of a RNA polymerase II promoter. Downstream of a T7 promoter, the minigenome plasmid contains the 5′ and 3′ non-coding regions (NCR) of a CCHFV genomic segment (S, M, or L) flanking a gene encoding a reporter protein (NanoLuc) in the negative orientation. Transfection of the helper plasmids yields the corresponding proteins to enable transcription of the minigenome plasmid and production of minigenome-derived vRNA. Following T7 transcription, the vRNA is encapsidated to form the genomic RNP. The RNP is subsequently transcribed into mRNA (secondary transcription) and translated to yield the reporter protein or replicated to produce additional vRNA. vRNA generated by both T7 transcription and RNA replication can be used as templates for transcription of reporter gene mRNA by NP and the L protein, resulting in enhanced reporter activity. A measurable luminescent signal is produced by hydrolysis of an externally provided reporter substrate.

The minigenome system is useful for studying viral transcription, replication, and encapsidation using model RNAs. Instead of a full-length vRNA or cRNA, it uses genome or antigenome analogues called minigenomes. The minigenome contains the terminal NCRs of a genomic segment, but the CCHFV coding region is replaced with a reporter gene [25,26,27]. Since the minigenome system is non-infectious and does not use full CCHFV genomes, it allows the study of CCHFV outside of high biocontainment laboratories. In the minigenome system, DNA copies of the vRNA minigenomes are cloned into expression vectors under the control of a T7 promoter; co-transfection with a T7-encoding plasmid yields a minigenome RNA (Figure 5). As with other reverse genetics systems that rely on T7 promoter expression, a single terminal G is added to the 5′ RNA termini to enhance T7 activity, and a native 3′ terminus is generated using a hepatitis delta virus ribozyme [56]. The NP and L protein are provided either from expression plasmids or by superinfection with CCHFV. The minigenome RNA is encapsidated, and acts as a template for replication and for transcription, resulting in the production of mRNA and ultimately a reporter signal. The reporter signal provides a quantitative collective measure of genome replication, transcription and translation [112,113]. However, by using an L protein with a mutated catalytic D693 it is possible to monitor replication alone as this mutant is unable to transcribe mRNA due to a lack of cap snatching activity [25]. While highly useful, the CCHFV minigenome system is limited as it does not model all aspects of the viral replication and cannot be used to study processes requiring the glycoproteins, such as entry, virus assembly, and egress.

### 4.2. CCHFV Virus-like Particle System

**Figure 6:**
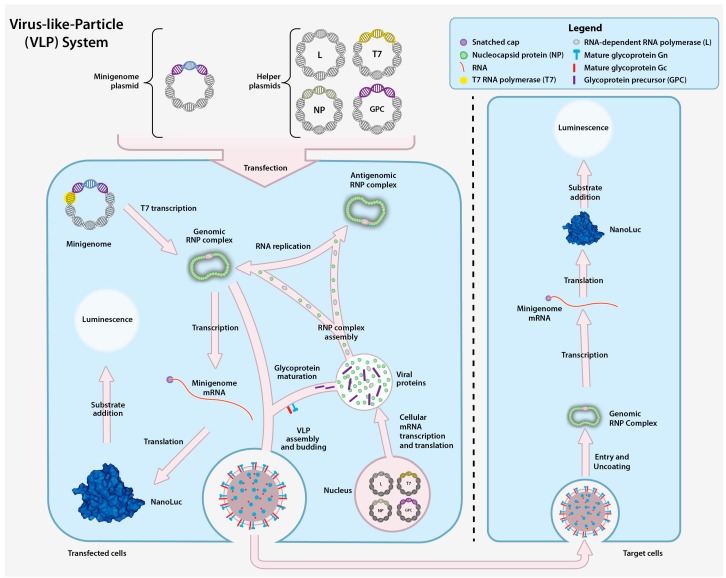
Transcriptionally and entry competent CCHF Virus-like-Particle (tec-VLP) system. The VLP system is composed of the minigenome plasmid and four helper plasmids that collectively encode the T7 polymerase, CCHFV NP, L, and GPC downstream of a cellular RNA polymerase II promoter. The corresponding proteins facilitate the generation of minigenome plasmid-derived vRNA. The minigenome plasmid is transcribed by T7 and the resulting vRNA is encapsidated to form the genomic RNP. The genomic vRNA is amplified from antigenomic RNPs, and subsequently transcribed and translated into the reporter protein. Reporter activity is monitored by adding a luciferase substrate and measuring the luminescent signal. VLPs assemble and bud from transfected cells and can subsequently enter other cells. Upon release of the genomic RNP from the VLP in the recipient cells, the minigenome can be amplified and transcribed into reporter mRNA, resulting in reporter protein expression.

In order to overcome some of the limitations inherent to the minigenome system, entry competent virus-like particle (VLP) systems have also been developed. A VLP contains all viral proteins and a minigenome. Therefore it is unable to express viral proteins upon entering a target cell. The particles in these systems can mimic a single-cycle infection; and because they do not encode CCHFV proteins, this system can be studied outside of high biocontainment laboratories. VLPs are generated by expressing three helper plasmids encoding the NP, GPC, and L protein together with a minigenome plasmid, resulting in the incorporation of encapsidated minigenome RNA into VLPs (Figure 6). The VLPs are able to enter target cells, and the RNPs can serve as templates for replication and transcription when the CCHFV NP and the L protein are supplied in trans. To date, two different CCHFV VLP systems have been developed [25]. In the first system, transcriptionally competent VLPs (tc-VLP) are incubated with target cells expressing the CCHFV NP and L protein in order to obtain robust *Renilla* reporter activity that can be used to study cell entry [25]. In the second system, transcriptionally and entry competent VLPs (tec-VLPs) are generated using a codon-optimized version of the L protein and GPC, and a NanoLuc reporter to produce VLPs. The resulting tec-VLPs can enter target cells to generate a robust NanoLuc signal without the need to express L protein and NP in the target cells, simplifying workflow for studying entry and primary transcription (Figure 6) [27].

### 4.3. CCHFV Infectious Clone System

**Figure 7:**
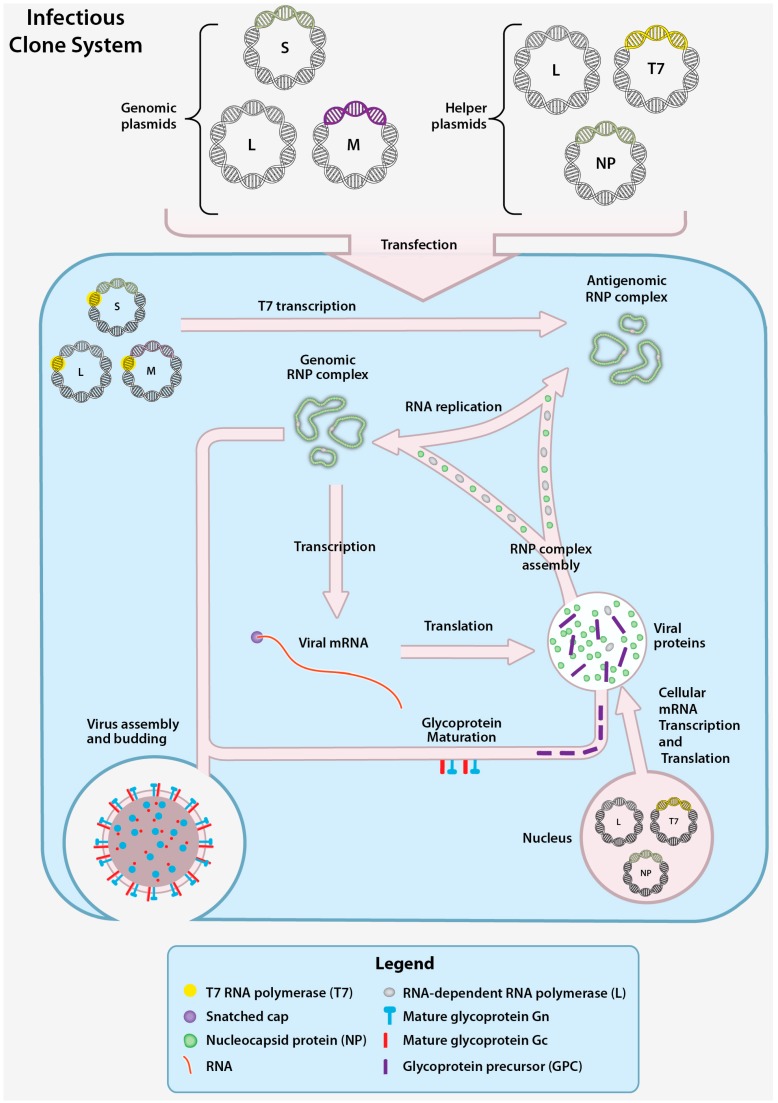
CCHFV infectious clone system. The CCHFV infectious clone system functions by co-transfection of genomic plasmids and helper plasmids into cells. The genomic plasmids each contain the DNA sequence of one of the CCHFV segments (S, M, or L) in the positive sense orientation downstream of a T7 promoter. The three helper plasmids encode the T7 RNA polymerase, the CCHFV NP and the L genes downstream of cellular RNA polymerase II promoters. Following T7 transcription of the genomic plasmid (enabled by the T7 helper plasmid), the cRNA is encapsidated by NP and L protein to form the antigenomic RNP, and is subsequently replicated by helper plasmid-derived NP and L protein. The vRNAs from the genomic RNPs are transcribed to mRNA and translated to yield CCHFV mRNA. The CCHFV mRNA are translated into additional NP, L, and GPC. The GPC undergoes processing and to yield the mature Gn and Gc. Following the accumulation of genomic RNPs and mature glycoproteins, infectious CCHFV particles assemble and are released.

The CCHFV infectious clone system allows the production of infectious recombinant CCHFV (rCCHFV) from DNA plasmids and contains the complete genome sequence under the control of a T7 promoter. Successful rescue of rCCHFV requires the addition of helper plasmids and transfection into cells. In the rCCHFV rescue process three plasmids, each containing the full sequence of one of the genome segments, are transfected into permissive cells (e.g., Huh7, BSR-T7/5). The genome is then transcribed by T7 into cRNA copies (Figure 7) that are used as a replication template for vRNA. Helper plasmids designed to produce NP and L proteins are provided in trans to synthesize and encapsidate vRNA, and finalize production of reconstituted genomic RNPs. After genomic RNPs are obtained, replication and transcription can be driven by NP and L protein produced from viral mRNA. The production of all viral proteins (NP, GPC and L protein) ultimately assembles into rCCHFV particles capable of infecting neighboring cells and performing multiple infection cycles. Since rCCHFV is infectious, all precautions and biosafety level restrictions associated with live CCHFV experimentation must be adhered to. A key to the success of this system is codon optimization of the DNA sequence of the L helper plasmid. Codon optimization of the L protein increases the activity and the amount of full-length L protein present in transfected cells [56].

While the infectious clone system is the most comprehensive model available of the virus replication cycle, the newly generated rCCHFV must functionally perform all the basic steps of the viral replication cycle for successful rescue of virus. Mutations that abolish a protein function that is critical for replication will prohibit virus rescue without insight into which specific aspects of the replication cycle have been disrupted. Minigenome and VLP systems can be used to more precisely dissect the effects of mutations or treatments on the individual steps in the viral replication cycle.

## 5. Conclusions and Future Directions

CCHF is a medically important tick-borne disease with a wide endemic distribution. At present, antiviral strategies to treat human CCHFV infection remain controversial or experimental. The most widely used antiviral, Ribavirin, has been shown to be effective against CCHFV *in vitro* and in animal models, but its clinical benefit remains unproven [114,115,116]. Although significant advances have been made in the field in recent years, many aspects of the CCHFV replicative cycle and pathogenesis still remain poorly defined. Additional studies to improve our knowledge of all viral proteins are warranted. Structural and/or sequence similarities of the CCHFV NP to the NP of related, better-studied arenaviruses may help to predict the putative functions of the CCHFV NP domains and guide future research. The advent of more advanced molecular tools to study NP in the context of a viral infection has the potential to greatly enhance our understanding of the roles of NP and the potential differences between various CCHFV genotypes.

In addition, studying CCHFV glycoprotein function is a critical area of research due to the significant gaps in our knowledge of the essential roles of the glycoproteins in the viral replication cycle. Improved knowledge of glycoprotein processing may provide targeted development of medical countermeasures. Since furin has a relatively minor role in the CCHFV replication cycle compared to SKI-1/S1P, pharmacological inhibitors of SKI-1/S1P may represent a promising drug target. Efficacy studies of proprotein convertase inhibitors *in vivo* and *in vitro* are critically needed to better assess the therapeutic potential of furin and SKI-1/S1P inhibition.

Further development of our understanding of the OTU domain will provide key information about CCHFV-mediated innate immune modulation, and OTU-defective or inactive mutants may be potential vaccine candidates. Reverse genetics can be used to investigate CCHFV OTU mutants that selectively cleave Ub or ISG15 conjugates, shedding light on the specific roles these conjugates play during CCHFV infection. Future experiments should focus on investigating OTU function in the context of virus infection. Previous experiments overexpressing the CCHFV OTU domain have provided valuable insights into its function, but may not accurately reflect events during a natural infection, where other factors, like spatiotemporal distribution, may affect the full-length L protein and OTU domain activity.

Advancement in all the aforementioned areas should be complemented and supported by additional investigation into viral interactions with the tick-vector and host. Studies on the molecular aspects of CCHFV replication are almost exclusively focused on cell culture systems of mammalian cells. Since CCHFV establishes a persistent infection in ticks, CCHFV replication and viral protein function should be studied in ticks and tick cell lines [117,118]. Additional research involving the tick vector and vector derived cells may aid in elucidating aspects of CCHFV that remain unclear in traditional mammalian cell based studies.

Existing interferon-α/β receptor (IFNAR)^−/−^ and STAT1^−/−^ mouse models of CCHF have been used to screen the efficacy of antivirals [119,120] and experimental vaccines [121,122,123,124]. The development of the existing disease models and studies of CCHFV-infected cells indicate the importance of the early innate immune response in disease [88,125]. In order to better model early events in human infection, continued efforts should be made in the development of immunocompetent CCHF animal models. An immunocompetent model may provide critical insight into correlates of protection and enhance our understanding of the molecular mechanisms underlying pathogenesis that are largely restricted to *in vitro* investigations at this time. Given the complex role of the immune response in CCHF pathogenesis, such models would be invaluable for screening new therapeutic and vaccine candidates.

CCHFV has been recognized as a significant emerging public health threat and is becoming a higher public health priority. Vaccine and therapeutic development for CCHF are rapidly expanding fields. However, a summary of these fields is beyond the scope of this review. Here we review recent *in vitro* experimental research efforts aimed at elucidating the mechanisms of CCHFV replication and pathogenesis. We provide a comprehensive summary of the known aspects of the CCHFV replication cycle, viral protein function, and cell-mediated host responses to infection. We also identify areas in CCHFV research that remain unknown or unclear, and merit additional investigation.
